# Cancer Evolution in Precision Medicine Era

**DOI:** 10.3390/cancers14081885

**Published:** 2022-04-08

**Authors:** Dimitris Karagiannis, Theodoros Rampias

**Affiliations:** 1 Department of Genetics and Development, Columbia University Medical Center, New York, NY 10032, USA; 2 Biomedical Research Foundation of the Academy of Athens, 11527 Athens, Greece

Recent advances in our understanding of cancer, driven mainly by the emergence of new technologies have highlighted that heterogeneity shapes not only the genetic profile of tumors but also their epigenetic and gene expression profile. Moreover, the emerging picture supports a view of cancer which goes beyond the genome of tumor cells and emphasizes the level of cell-tissue interface. In this framework, communication between different tumor subclones as well as between tumor subclones and stroma cells plays a crucial role in tumor evolution. Deregulated epigenome and altered gene expression in tumor subclones generate different cellular states and increase the phenotypic plasticity of cancer cells. During tumor evolution, this plasticity promotes adaptation to selection pressures driven by different factors such as the immune system, mechanical stress, metabolic and oxygen supply stress, and drug treatment (Figure 1). In this context, there is a growing appreciation that non-genetic heterogeneity contributes to treatment failure and confers to drug resistance [1,2]. This special issue covers cancer research topics related to the fast-moving field of tumor evolution ranging from characterization of tumor heterogeneity to therapeutic targeting of tumor heterogeneity. We hope this special issue will provide a comprehensive picture of recent research activities in this field.

## 1. Genetic and Non-Genetic Heterogeneity

Variability in the genetic context of tumor cells is a major mechanism of resistance to therapy and cancer recurrence. Siraj and colleagues profiled approximately 200 colorectal tumors by whole exome sequencing, that were either therapy-naive primary, treated primary or metastases. This approach revealed how genetic diversity affected therapy outcome, and conversely how therapy affected genetic diversity in the primary tumors and metastases. Among other findings, they identified actionable mutations in primary tumors and metastases that could improve clinical outcome [3]. Kanaki and colleagues established a biobank of Non-Small Cell Lung Cancer Patient-Derived Xenografts in order to study tumor evolution in conjunction with patient epidemiology, clinical progression, genetic make-up and circulating tumor cell characterization. This work detected clinical and genetic factors that affect grafting potential, as well as notable genetic differences between PDXs and corresponding primary tumors, which is often not considered in PDX studies [4].

Heterogeneity in gene expression can influence the extracellular environment and alter metastatic potential. Gant and colleagues used Second Harmonic Generation (SHG) microscopy, machine learning and mass spectrometry to examine collagen architecture in high grade serous ovarian cancer (HGSOC). Their methodology uncovered differential collagen isoform expression during disease progression that is associated with changes in the collagen fiber and extracellular matrix (ECM) morphology [5]. In breast cancer, Gerashchenko and colleagues identified a new type of invasive torpedo-like structure, and through molecular characterization found that expression of KIF14 and Mieap and absence of EZR at cells at the tip of the structure is associated with distinct metabolism and chemokine signaling. In addition, patients with this biomarker displayed increased frequency of metastases and worse metastasis-free survival [6]. Lee and colleagues surveyed a cohort of primary lung adenocarcinoma tumors and brain metastases for expression of epigenetic and EMT factors. Their work revealed high expression of EMT markers in brain metastasis and strong positive correlation of MML4 with SLUG, UTX with ZEB1, and negative correlation between EZH2 and TWIST [7]. 

Cancer cells can adapt their metabolism to stress based on their genetic background. This type of metabolic plasticity is associated with heterogeneity and resistance to treatment. Interestingly, Mostazo and colleagues identified a link between metabolic plasticity and resistance to tyrosine kinase inhibitors (TKIs) in chronic myeloid leukemia (CML) [8]. TKI-resistant CML cells displayed pronounced metabolic reprogramming, specifically increased glycolysis, fatty acid synthesis and TCA cycle. Both TKI-naïve and -resistant cells were sensitive to inhibition of serine-glycine one carbon metabolism and could be considered for management of TKI resistance.

## 2. Tumor Microenvironment (TME)

The TME is rich in various types of immune and stroma cells that interact with the tumor and influence its progression and response. The advancements in cancer immunotherapy have underscored the importance of innate immunity for therapy. HNSCC tumors are characterized by inflammation and immune cell infiltration. Economopoulou and colleagues review how HNSCC tumor cells interact with other TME cell populations (e.g., CAFs, TAMs, MDSCs, Tregs) to escape the immune system, as well as the ongoing efforts to improve response to therapy by stimulation of immune cells through immunotherapy and antiangiogenics [9].

Signals from the TME influence cancer cell biology in a localized manner and thus promote tumor heterogeneity. Brunn and colleagues found a novel link between the interferon inducible gene IRF9 and increased tumor growth and metastasis in lung adenocarcinoma cell lines through transcriptional regulation of the ECM protein versican (VCAN) [10]. Novotný and colleagues investigated the complex interaction between melanoma and stromal cells, by generating heterogeneous melanoma spheroids. Using single-cell RNA sequencing, they identified heterogeneity in tumor-associated fibroblasts, which is associated with photodamage and melanoma invasiveness [11]. An emerging research field investigates the role of nerves in tumor progression and therapy. Recent research has shown significant innervation in many tumors demonstrating that signals from nerve cells can promote cancer growth and vice versa. Schonkeren et al summarized how components of the gut nervous system interact and influence colorectal tumors. That influence can be negative or positive depending on the context, which therefore must be considered when designing therapeutic approaches [12].

## 3. Biomarkers

Biomarkers provide valuable prognostic information and can guide clinical course of action. Moreover, the identification and characterization of biomarkers can improve our appreciation of tumor heterogeneity and guide therapy. In this issue, several novel types of biomarkers were reported. Zavridou and colleagues profiled circulating tumor cells (CTCs) and exosomes in metastatic castration resistant pancreatic cancer (mCRPC) patients for biomarkers that can inform clinical management (e.g., AR-V7 expression). They identified significant associations of worse overall survival with gene expression in CTCs (e.g., CK19, TWIST1) and with DNA methylation in exosomes (e.g., GSTP1) [13]. Recent studies have demonstrated that tRNA-derived fragments (tRFs) function as non-coding RNAs (ncRNAs). In this context, Papadimitriou and colleagues identified 5′-tRF-LysCTT expression to be altered in bladder cancer and associated with tumor aggressiveness, early disease progression and poor treatment outcome [14]. Cancer-specific circulating autoantibodies are an emerging type of biomarker. Jong and colleagues highlight recent advances in detection of autoantibodies for cancer detection and prognosis [15]. Further research in autoantibodies could improve patient stratification as well as development of new immunotherapy treatments. To disentangle tumor heterogeneity in cardiac sarcoma, a rare, understudied disease with limited treatment options, Geronikolou and colleagues employed molecular networking to construct interaction networks for each type of primary heart sarcoma and thus identified key gene nodes that are associated with distinct sarcoma types and clinical symptoms [16].

## 4. Prevalence and Management of Tumor Heterogeneity

Heterogeneity presents a major obstacle for the treatment of most types of cancer. Shlyakhtina and colleagues reviewed current literature on models of clonal evolution during cancer development [17]. Their work highlights the models of neutral and branching evolution as the most prevalent ones in the majority of diseases. This is supported by reports indicating high levels of genetic and non-genetic heterogeneity observed in most cancers. In the context of therapy, they propose redirection of cancer evolution as a therapeutic approach. Baliu-Pique and colleagues reviewed recent literature on how phenotypic features of intratumoral heterogeneity in breast cancer can hinder effective treatment [18]. They conclude that future personalized therapies should rely on drug combinations to mitigate emergence of resistant populations. Using comparable rationale, we propose ways that HDAC inhibitors can be utilized in various cancer contexts to reduce heterogeneity in gene expression, metabolism, plasticity or the micro-environment to enhance therapeutic efficacy [19]. 

## 5. Conclusions

In conclusion, this special issue includes a wide collection of publications that present novel methodologies, findings and perspectives that contribute to our understanding of tumor heterogeneity. Highlights include development of new models and tools for tumor studies, discovery of genetic interactions and biomarkers and thought-provoking insights on future therapeutic avenues. 

## Figures and Tables

**Figure 1:**
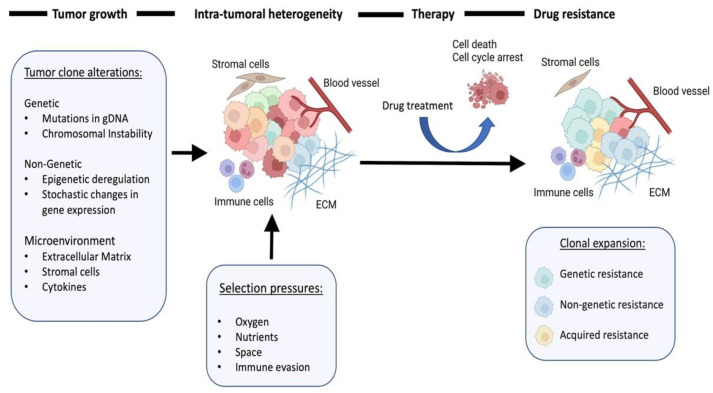
Simplified illustration depicting how intra-tumoral heterogeneity is shaped during cancer progression and treatment. During tumor growth, cells are subject to forces that promote clonal variation (Tumor clone alterations) or reduce it (Selection pressures). Upon treatment, most tumor cells undergo cell death, leading to expansion of resistant clones.
